# Phytoceramide Shows Neuroprotection and Ameliorates Scopolamine-Induced Memory Impairment

**DOI:** 10.3390/molecules16119090

**Published:** 2011-10-28

**Authors:** Jae-Chul Jung, Yeonju Lee, Sohyeon Moon, Jong Hoon Ryu, Seikwan Oh

**Affiliations:** 1 Department of Neuroscience and TIDRC, School of Medicine, Ewha Womans University, Seoul 158-710, Korea; 2 Department of Oriental Pharmaceutical Science, College of Pharmacy, Kyung Hee University, Seoul 130-701, Korea; 3 Institute of Life Science Research, Rexgene Biotech, Ochang, Chungbuk 368-831, Korea

**Keywords:** phytoceramide, phytosphingosine, neurotoxicity, memory, L-glutamate, neuron

## Abstract

The function and the role phytoceramide (PCER) and phytosphingosine (PSO) in the central nervous system has not been well studied. This study was aimed at investigating the possible roles of PCER and PSO in glutamate-induced neurotoxicity in cultured neuronal cells and memory function in mice. Phytoceramide showed neuro-protective activity in the glutamate-induced toxicity in cultured cortical neuronal cells. Neither phytosphingosine nor tetraacetylphytosphingosine (TAPS) showed neuroproective effects in neuronal cells. PCER (50 mg/kg, p.o.) recovered the scopolamine-induced reduction in step-through latency in the passive avoidance test; however, PSO did not modulate memory function on this task. The ameliorating effects of PCER on spatial memory were confirmed by the Morris water maze test. In conclusion, through behavioral and neurochemical experimental results, it was demonstrated that central administration of PCER produces amelioration of memory impairment. These results suggest that PCER plays an important role in neuroprotection and memory enhancement and PCER could be a potential new therapeutic agent for the treatment of neurodegenerative diseases such as Alzheimer’s disease.

## 1. Introduction

The major pathogenic mechanisms of neuronal damage include excitotoxicity, production of reactive free radicals, inflammation, and programmed cell death (apoptosis) [1]. Activation of glutamate receptors, through the attendant failure of ion homeostasis and increase in intracellular Ca^2+^ concentration, is a major factor involved in initiating ischemic cell death [2]. A straightforward therapeutic approach, therefore, is to block the receptors that are activated by glutamate.

It has been known that brain cholinergic system losses are closely associated with the cognitive deficits observed in Alzheimer’s disease [3]. It is well documented that the selective inhibition of central acetylcholine esterase might increase acetylcholine neurotransmission in the synaptic cleft of brain, resulting in improvements in cognitive function [4]. An agent that can ameliorate cognitive dysfunction and neurotoxocity induced by glutamate or cholinergic dysfunction should be effective in the treatment of various neurodegenerative diseases, including Alzheimer’s disease. The scopolamine-induced amnesic animal model has been used to screen for potential treatments for cognitive dysfunction [5].

In regard to brain functions, major findings have emphasized the significance of sphingolipids as bioactive molecules that control diverse cellular processes such as proliferation, differentiation, growth, senescence, migration, and apoptosis [6]. Sphingolipid metabolites such as ceramide and sphingosine 1-phosphate (S1P), have received much attention as key regulators of cell death and survival [7]. Ceramide mediates a wide array of the stress signals leading to growth arrest or cell death, whereas S1P exerts prosurvival capabilities by antagonizing ceramide effects [8]. The major sphingoids of mammalian cells are sphingosine and dihydrosphingosine. In the sphingolipid pathway, sphingosine is phosphorylated to form S1P by the sphingosine kinases. Because the phosphorylation of sphingosine is the only pathway for the formation of S1P, cellular S1P is highly dependent on the availability of sphingosine generated by ceramidases.

**Figure 1:**
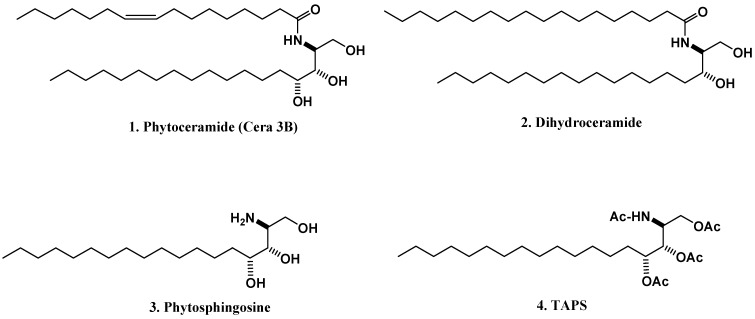
Structures of phytoceramide (**1**), dihydroceramide (**2**), phytosphingosine (**3**), and TAPS (**4**).

A very long chain fatty acyl-CoA is linked by an amide bond to dihydrosphingosine or phytosphingosine (PSO) to generate dihydroceramide or phytoceramide (PCER, Figure 1) [9]. Dihydroceramide is hydroxylated at C4 to yield PSO, which is the primary sphingoid base bound in most fungal and plant ceramides [9]. Phytosphingosine (Figure 1) is acylated by various fatty acids to form phytoceramides. In yeast cells, dihydroceramide is mainly hydroxylated to form PSO, which is acylated by various fatty acids to form PCER [10], suggesting that PSO can be synthesized *de novo* in yeast cells. Phytosphingolipids commonly possess amide and alcohol groups on the sphingosine backbone (Figure 1). Phytoceramides can be hydrolyzed to form PSO through the action of a ceramidase [11] indicating that, like sphingosine, PSO may only be generated from the hydrolysis of PCERs and not from *de novo* biosynthesis in mammalian cells. These observations suggest that the generation of sphingosine or PSO in mammalian cells is only controlled by ceramidases. Ceramide is at the centre of sphingolipid metabolism and has been recognized as a critical second messenger.

Profound losses in the cholinergic system of brain are closely associated with the cognitive deficits observed in Alzheimer’s disease [3]. Therefore, the current study focused on the effects of the PCER and PSO on memory impairment by suppression of cholinergic activity in the central nervous system of mice. The memory performance was examined by using passive avoidance and Morris water maze tests in mice whose memory was impaired by scopolamine treatment.

## 2. Results and Discussion

### 2.1. Neuroprotection in Cultured Neuronal Cells

**Figure 2:**
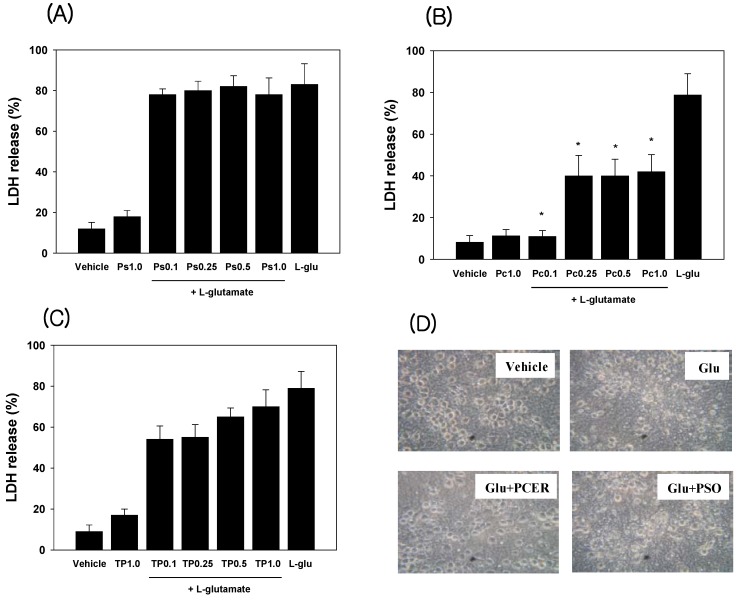
Protection of glutamate-induced neurotoxicity in cultured cortical neurons with phytoceramide. Sphingolipids were co-treated with glutamate (60 µM) in cultured neuronal cells for 24 h at 37 °C. Lactate dehydrogenase (LDH) was measured at 340 nm using a microplate spectrophotometer. Data represent the mean ± standard error (SE). * *p* < 0.05 in comparison with L-glu treated group. Ps: phytosphingosine; Pc: phytoceramide; TP: TAPS.

Lactate dehydrogenases (LDH) are valuable *in vitro* markers for cellular toxicity [12]. In these studies, LDH activity was used as a measure of the neuroprotective effects of the sphingolipids. Compounds were added to the culture medium with glutamate, and neuroprotection was observed via microscopic images and measuring the glutamate-induced LDH release. Cultured neurons without glutamate or with sphingolipid alone did not show neurotoxicity, while glutamate (60 µM) induced neurotoxicity. Phytoceramide showed the neuroprotective activity in glutamate treated cortical neuron cells (Figure 2).

The level of LDH release by glutamate treatment was decreased with phytoceramide (1, 0.25, 0.5, 1.0 µM). However, phytosphingosine or TAPS did not exhibit anti–neurotoxic activities at the same dose as phytoceramide treatment.

### 2.2. Amelioration of the Scopolamine-Induced Memory Deficits in the Passive Avoidance Test

We tested whether PCER virtually caused the behavioral change on memory in the passive avoidance test. Scopolamine-induced memory deficits during the passive avoidance test are dependent on long-term memory [13].

**Figure 3:**
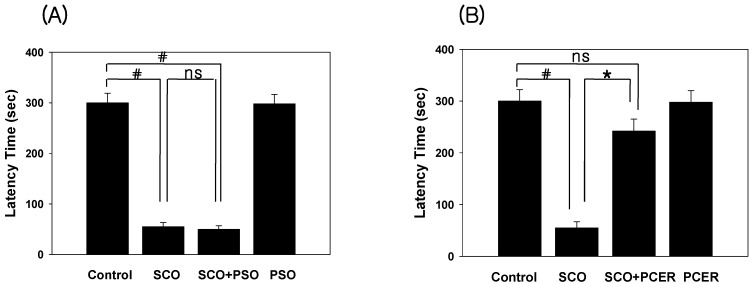
Effects of PSO and PCER on the scopolamine-induced memory deficits in the passive avoidance test. Memory impairment was induced by scopolamine (SCO) treatment (1 mg/kg, i.p.). At 60 min before acquisition trials, PSO (50 mg/kg), PCER (50 mg/kg) or vehicle (saline) was administered orally (p.o.) to mice. Eight different animals were used in each treatment group. Acquisition trials were carried out 30 min after a single scopolamine treatment. At 24 h after acquisition trials, the retention trials were carried out for 5 min. Data were analyzed by two-way ANOVA for multiple comparison. Data represent the mean ± standard error (SE). ^#^* p *<0.05 in comparison with control, * *p* < 0.05 in comparison with scopolamine treated group. NS: non significance

When the effect of PCER on scopolamine-induced memory deficits was tested using the passive avoidance test (Figure 3), Oral administration of PCER (50 mg/kg, p.o.) ameliorated the scopolamine-induced memory impairment in step-through latency (*p* < 0.01); however, PSO did not modulate the memory function. During the acquisition trial, no differences in latencies were observed among the treated groups.

### 2.3. Amelioration of the Scopolamine-Induced Memory Deficits in Morris Water Maze Test

**Figure 4:**
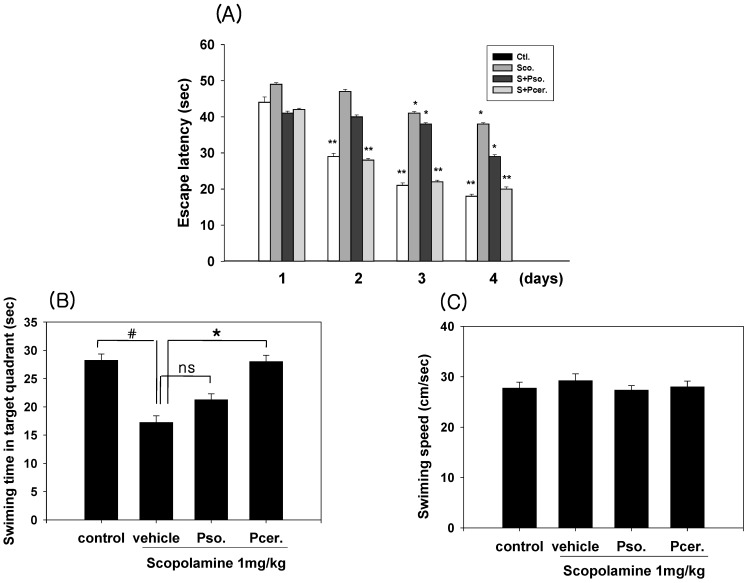
Effects of PSO and PCER on the escape latencies in the training trial sessions and on the swimming time in the probe trial session of the Morris water maze task in scopolamine-induced memory deficit mice. PSO, PCER (50 mg/kg) or vehicle (saline) was orally administered to mice at 60 min before training trial session. Memory impairment was induced by scopolamine (1 mg/kg) intraperitoneal treatment. Eight different animals were used per each treatment group. The training trial and the probe trial sessions were performed as described in Materials and Methods. Data represent the mean ± standard error (SE). ^#^* p* < 0.05 in comparison with control, * *p* < 0.05 in comparison with treated group. NS: non significance.

The effects of phytoceramide (50 mg/kg, p.o.) on spatial learning and memory were evaluated by the Morris water maze test. The scopolamine-treated group exhibited longer escape latencies throughout the training days than those of the control group. PCER significantly shortened the escape latencies which were prolonged by scopolamine treatment (*p* < 0.01). In the probe trial sessions, swimming times within the platform quadrant for the scopolamine-treated group were significantly lower than those of the vehicle-treated normal group animals (*p* < 0.01) (Figure 4). Moreover, the swimming time shortened by scopolamine within the platform quadrant was significantly increased by PCER (*p* < 0.05), but not by PSO, although no significant differences in swimming speeds were observed between the groups. Ceramide has been known as a sphingolipid with potent proinflammatory and proapoptotic properties [14,15,16] and its active metabolite, ceramide 1-phosphate, stimulates macrophage proliferation through activation of the PI3-kinase, JNK and ERK pathways [17]. However, studies using primary cultures of neurons demonstrated that ceramide has multiple functions, depending on the cell type and the developmental stage.

In immature hippocampal neurons, ceramide plays bipotential roles in cell survival and dendrite outgrowth in a dose-dependent manner [18]. It was also found that ceramide prevents cell death of motoneurons cultures through inhibition of oxidative signals [19]. These results suggest that the cellular level of ceramide is critical for regulation of neuronal survival and differentiation. In our results PCER inhibited the glutamate-induced neurotoxicity in cultured neuronal cells, while PSO did not show the neuroprotective effect with same dose of PCER in the cultured neuronal cells. There are some structural differences between PCER and PSO. Phytosphingosine is structurally similar to sphingosine, except that PSO has a hydroxyl group at C-4 of the sphingoid long-chain base instead of the *trans* double bond between C-4 and C-5.

Scopolamine, which is a muscarinic receptor antagonist, can cause amnesia in animals by blocking cholinergic neurotransmission and impairs learning and memory in rodents and humans. It was determined whether PCER attenuates scopolamine-induced learning and memory impairments through the passive avoidance, Y-maze test, and water-maze tests. PCER reversed the memory deficits induced by scopolamine. The passive avoidance test is a useful tool for the estimation of standard learning and memory. Then, this test has been used as an indicator of short-term and long-term memory [20].

In the memory test, scopolamine increased the escape latency time at the training sessions. PCER shortened this escape latency time on day 4. In the probe trial session, PCER increased the swimming time within the target quadrant. The decrease in escape latency from day to day in the first trial represents long-term memory or reference memory [21]. The time in the quadrant with the platform reflects changes in spatial memory [22]. These results suggest that PCER improves the long-term memory in amnesic mouse models induced by scopolamine treatment. The group with PCER was recovered from memory deficit while the group with PSO did not recover.

## 3. Experimental

### 3.1. Materials and Methods

Male ICR mice (28–30 g) were purchased from Orient Labanimal (Seoul, Korea). Mice allowed access to water and food *ad libitum* were grouped 5–6 per cage, and maintained at an ambient temperature of 23 °C and a 12 h diurnal light cycle (light on 07:00–19:00). All behavioral experiments were carried out in a room adjacent to that in which the mice were housed under the same conditions of temperature and light cycle. All the experiments were carried out according to the guidelines of the Animal Care and Use Guidelines of Ewha Womans University, Korea. Scopolamine was obtained from Sigma-Aldrich (St. Louis, MO, USA).

Flash chromatography was carried out on silica gel 60 [Scientific Adsorbents Incorporated (SAI), particle size 32–63 *µ*m, pore size 60 Å]. ^1^H-NMR, and ^13^C-NMR spectra were recorded on a Bruker DPX 400, 500 at 400, 500 MHz and 100, 125 MHz; respectively. The chemical shifts are reported in parts per million (ppm) downfield from tetramethylsilane, and *J*-values are in Hz. Infrared (IR) spectra were obtained on an ATI Mattson FT/IR spectrometer. Mass spectra were recorded with a Waters Micromass ZQ LC-Mass system and high resolution mass spectra (HRMS) were measured with a Bruker BioApex FTMS system by direct injection using an electrospray interface (ESI).

Phytoceramide (**1**) and phytosphingosine (**3**) were provided by Doosan Glonet (Suwon, Korea) [23]. Those sphingolipids were readily characterized through after purification by flash column chromatography. *Phytoceramide* (**1**): R*_f_* = 0.4 (85:15:2; chloroform-methanol-ammoniumhydroxide 28%, *v*/*v*); IR (neat, NaCl) 3316, 2921, 2851, 1736, 1633, 1540, 1466, 1377, 1246, 1042, 721 cm^−1^; ^1^H-NMR (400 MHz, CDCl_3_) δ 5.30 (t, 2H, *J* = 5.60 Hz, C*H*=C*H*), 4.67 (s, 3H, 3 × O*H*), 4.06 (dd, 1H, *J* = 4.80, 4.80 Hz, C*H*-NH), 3.76–3.64 (m, 2H, C*H*_2_OH), 3.55-3.40 (m, 2H, 2 × C*H*OH), 3.36–3.27 (m, 1H, N*H*), 2.19 (t, 2H, *J* = 7.60 Hz, C*H*_2_CO), 2.04–1.89 (m, 4H, C*H*_2_CH=CHC*H*_2_), 1.68–1.46 (m, 4H, C*H*_2_CH_2_, C*H*_2_CHOH), 1.43–1.18 (m, 44H, 22 × C*H*_2_), 0.85 (t, 6H, *J* = 6.80 Hz, 2 × C*H*_3_); ^13^C-NMR (100 MHz, CDCl_3_) δ 175.49, 130.51, 130.32, 75.86, 73.00, 61.76, 52.74, 37.01, 33.15, 32.58, 32.56, 30.49, 30.35, 30.29, 30.19, 30.15, 30.08, 30.00, 29.98, 29.96, 29.93, 29.91, 29.81, 27.78, 26.55, 26.53, 23.28, 14.36; MS calcd. for C_36_H_71_NO_4_Na: 604.52 [M+Na]^+^, found: 604.58.

*Phytosphingosine* (**3**): White solid. mp 101 °C; R*_f_* = 0.2 (85:15:2; chloroform-methanol-ammonium hydroxide 28%, *v*/*v*); IR (neat, NaCl) 3454, 2917, 2850, 1635, 1376, 1072, 838 cm^−1^; ^1^H-NMR (400 MHz, CDCl_3_) δ 5.17 (br s, 4H, N*H*, 3 × O*H*), 3.90 (d, 1H, *J* = 10.40 Hz, C*H*_2_OH), 3.75 (t, 1H, *J* = 7.60 Hz, C*H*_2_OH), 3.55–3.40 (m, 2H, 2 × C*H*OH), 3.36–3.24 (m, 2H, C*H*NH, N*H*), 1.82–1.69 (m, 1H, C*H*_2_CH_2_OH), 1.62–1.42 (m, 1H, C*H*_2_CH_2_OH), 1.40–1.16 (m, 24H, 12 × C*H*_2_), 0.85 (t, 3H, *J* = 6.80 Hz, C*H*_3_); ^13^C-NMR (100 MHz, CDCl_3_) δ 73.26, 73.04, 58.70, 56.14, 35.12, 32.78, 30.57, 30.51, 30.48, 30.19, 26.62, 26.05, 23.46, 14.34; MS calcd. for C_18_H_40_NO_3_: 318.30 [M+H]^+^, found: 318.16; [M+Na]^+^, found: 340.31.

### 3.2. Cortical Neuronal Cell Culture

Cortical cell cultures were prepared from ICR mice embryos at a gestational age of 15 days. The cortex was dissected and kept in an ice-cold solution. The cortical tissues were dissociated to single cells by gentle suspension. The cell suspension was centrifuged at 1,000 rpm for 5 min, and the resulting pellets were resuspended in minimal essential media (MEM), supplemented with 5% heat-inactivated fetal calf serum, mouse serum, glutamine, and glucose. The cells were plated on plates coated with poly-D-lysine and laminin at a density of 4.8 × 10^5^ cells/well in 24-well cultured plates. The cells were cultured in a CO_2_ incubator (5% [v/v], 37 °C). Seven days after plating, cells were treated with 10 µM cytosine arabinofuranoside (Ara C) to reduce the growth of contaminating non-neuronal cells. After treatment for 48 h, cells were fed with fresh media (without fetal calf serum).

### 3.3. Lactate Dehydrogenase (LDH) Assay

Lactate dehydrogenase released into the culture medium was measured by monitoring the production of NAD^+^ from NADH during the conversion of pyruvate to lactate. The cell supernatant (30 µL) was incubated in NADHP buffer (120 µL, 0.45 mg/mL), followed after 2 min by the addition of pyruvate (22 mM). The rate of NAD^+^ formation was monitored for 5 min at 11 s intervals at 340 nm by spectrophotometer.

### 3.4. Passive Avoidance Test

Passive avoidance test was carried out in identical illuminated and non-illuminated boxes (Gemini Avoidance System, San Diego, CA, USA). The illuminated compartment (20 × 20 × 20 cm) contained a 100 W bulb, and the floor of non-illuminated compartment (20 × 20 × 20 cm) was composed of 2 mm stainless steel rods spaced 1 cm apart. These compartments were separated by a guillotine door (5 × 5 cm). For the acquisition trial, mice were initially placed in the illuminated compartment and the door between the two compartments was opened 10 s later. When mice entered the dark compartment, the door closed and an electrical foot shock (0.25 mA) of 2.5 s duration was delivered through the stainless steel rods. One hour before the acquisition trial, mice were administered PSO or PCER (50 mg/kg, p.o.). Memory impairment was induced by scopolamine treatment (1 mg/kg, i.p.) 30 min after the administration of sphingolipid [24]. Control animals were administered saline only. Twenty four hours after acquisition trial, the mice were again placed in the illuminated compartment for the retention trials. The time taken for a mouse to enter the dark compartment after door opening was measured as latency times in both acquisition and retention trials. If a mouse did not enter the dark compartment within 300 sec, it was assumed that the mouse had remembered the single training trial.

### 3.5. Morris Water Maze Test

The Morris water maze is a circular pool (90 cm in diameter and 45 cm in height) with a featureless inner surface. The pool was filled to a depth of 30 cm with water containing 500 mL of milk (20 °C). The tank was placed in a dimly lit, soundproof test room with various visual cues. The pool was conceptually divided into quadrants. A white platform (6 cm in diameter and 29 cm high) was then placed in one of the pool quadrants and submerged 1 cm below the water surface so that it was invisible at water level. The first experimental day was dedicated to swimming training for 60 s in the absence of the platform. During the four subsequent days the mice were given four trials per day with the platform in place. When a mouse located the platform, it was permitted to remain on it for 10 s. If the mouse did not locate the platform within 60 s, it was placed on the platform for 10 s. The animal was taken to its home cage and was allowed to dry under an infrared lamp after each trial. The time interval between each trial was 30 s. During each trial, the time taken to find the hidden platform (latency) was recorded using a video camera-based Ethovision System (Nodulus, Wageningen, The Netherlands). For each training trial, mice were placed in the water facing the pool wall at one of the pool quadrants in a different order each day. One day after the last training trial sessions, mice were subjected to a probe trial session in which the platform was removed from the pool, allowing the mice to swim for 60 s to search for it. A record was kept of the swimming time in the pool quadrant where the platform had previously been placed. PSO (50 mg/kg, p.o.) or PCER (50 mg/kg, p.o.) was given 1 h before the first trial session at every consecutive day. Memory impairment was induced in mice with scopolamine (1 mg/kg, i.p.) at 30 min after treatment of the test agent [24]. Control group received saline only.

### 3.6. Statistical Analysis

The results were subjected to an analysis of the variance (ANOVA) using the Newman-Keuls multiple comparison test. Differences with * *P* < 0.05 and ** *P* < 0.01 were considered as statistically significant to analyze the difference.

## 4. Conclusions

In the present study it was observed that phytoceramide (PCER) showed neuroprotective effects in cultured neuronal cells and improves the memory impairment induced by scopolamine. However, phytosphingosine (PSO), which was produced from phytoceramide, did not affect the neurotoxicity or memory function. The ameliorating effect of PCER on memory impairment was confirmed in behavioral observation using the passive avoidance test and Morris water maze task. These results suggest that PCER could be applied as a therapeutic agent for enhancing the learning and memory functions.

## References

[1] Mergenthaler P., Dirnagl U., Meisel A. (2004). Pathophysiology of stroke: Lessons from animal models. Metab. Brain Dis..

[2] Richard M.J.P., Connell B.J., Khan B.V., Saleh T.M. (2011). Cellular mechanisms by which lipoic acid confers protection during the early stages of cerebral ischemia: A possible role for calcium. Neurosci. Res..

[3] Bartus R.T., Dean R.L., Beer B., Lippa A.S. (1982). The cholinergic hypothesis of geriatric memory dysfunction. Science.

[4] Dolezal V., Tucek S. (1991). Positive and negative effects of tacrin and methoxytacrine on the metabolism of acetylcholine in brain cortical prisms incubated under “resting” conditions. J. Neurochem..

[5] Ennaceur A., Meliani K. (1992). Effects of physostigmine and scopolamine on rats’ performances in object-recognition and radial-maze tests. Psychopharmacology.

[6] Elena I., de Chaves P. (2006). Sphingolipids in apoptosis, survival and regeneration in the nervous system. Biochim. Biophys. Acta.

[7] Spiegel S., Milstien S. (2003). Sphingosine-1-phosphate: An enigmatic signalling lipid. Nat. Rev. Mol. Cell Biol..

[8] Anne G.B., Dimitri P., Leyre B., Virginie G., Marie-Franc O.A., Marie-Lise M., Marie-Bernadette D., Olivier C. (2007). Critical role for sphingosine kinase-1 in regulating survival of neuroblastoma cells exposed to amyloid-β peptide. Mol. Pharmacol..

[9] Garcia J., Shea J., Alvarez-Vasquez F., Quershi A., Luberto C., Voit E.O., Del Poeta M. (2008). Mathmatical modeling of pathogenicity of Cryptococcus neoformans. Mol. Syst. Biol..

[10] Dickson R.C., Lester R.L. (1999). Yeast sphingolipids. Biochim. Biophys. Acta.

[11] Mao C., Xu R., Szulc Z.M., Bielawska A., Galadari S.H., Obeid L.M. (2001). Cloning and characterization of a novel human alkaline ceramidase: A mammalian enzyme that hydrolyzes phytoceramide. J. Biol. Chem..

[12] Koh J.Y., Choi D.W. (1987). Quantitative determination of glutamate mediated cortical neuronal injury in cell culture by lactate dehydrogenase efflux assay. J. Neurosci. Methods.

[13] Rubaj A., Zgodzinski W., Sieklucka-Dziuba M. (2003). The influence of adenosine A3 receptor agonist: IB-MECA, on scopolamine- and MK-801-induced memory impairment. Behav. Brain Res..

[14] Hannun Y.A. (1994). The sphingomyelin cycle and second messenger function of ceramide. J. Biol. Chem..

[15] Tang N., Ong W.Y., Zhang E.M., Chen P., Yeo J.F. (2007). Differential effects of ceramide species on exocytosis in rat PC12 cells. Exp. Brain Res..

[16] Cuzzocrea S., Paola R.D., Genovese T., Mazzon E., Esposite E., Crisafulli C., Bramanti P., Salvemini D. (2008). Anti-inflammatory and anti-apoptotic effects of fumonisin B1, an inhibitor of ceramide synthase, in a rodent model of splanchnic ischemia and reperfusion injury. J. Pharmacol. Exp. Ther..

[17] Gangoiti P., Granado M.H., Wang S.W., Kong J.Y., Steinbrecher U.P., Gomez-Munoz A. (2008). Ceramide 1-phosphate stimulates macrophage proliferation through activation of the PI3-kinase/PKB, JNK and ERK1/2 pathways. Cell Signal..

[18] Mitoma J., Ito M., Furaya S., Hirabayashi Y. (1998). Bipotential roles of ceramide in the growth of hippocampal neurons: Promotion of cell survival and dendritic outgrowth in dose- and developmental stage-dependent manners. J. Neurosci. Res..

[19] Irie F., Hirabayashi Y. (1999). Ceramide prevents motoneuronal cell death through inhibition of oxidative signal. Neurosci. Res..

[20] LeDoux J.E. (1993). Emotional memory systems in the brain. Behav. Brain Res..

[21] Morris R. (1984). Developments of a water-maze procedure for studying spatial learning in the rat. J. Neurosci. Methods.

[22] Blokland A., Geraerts E., Been M. (2004). A detailed analysis of rats’ spatial memory in a probe trial of a Morris task. Behav. Brain Res..

[23] Bae J.H., Sohn J.H., Park C.S., Rhee J.S., Choi E.S. (2003). Integrative transformation system for the metabolic engineering of the sphingoid base-producing yeast *Pichia ciferrii*. Appl. Environ. Microbiol..

[24] Park C.H., Choi S.H., Koo J.W., Seo J.H., Kim H.S., Jeong S.J., Suh Y.H. (2002). Novel cognitive improving and neuroprotective activities of *Polygala Tenuifolia* Willdenow extract, BT-11. J. Neurosci. Res..

